# Mutations Y493G and K546D in human HSP90 disrupt binding of celastrol and reduce interaction with Cdc37

**DOI:** 10.1002/2211-5463.12081

**Published:** 2016-05-25

**Authors:** Bin Peng, Yi‐Jun Gu, Ying Wang, Fan‐Fan Cao, Xue Zhang, Deng‐Hai Zhang, Jian Hou

**Affiliations:** ^1^ Department of HematologyChangzheng HospitalThe Second Military Medical UniversityShanghaiChina; ^2^ Sino‐French Cooperative Central LabShanghai Gongli HospitalThe Second Military Medical UniversityShanghaiChina; ^3^ National Center for Protein Science ShanghaiChina

**Keywords:** Cdc37, celastrol, heat shock protein 90, molecular modeling, protein drug interaction

## Abstract

Celastrol, a natural compound derived from the Chinese herb *Tripterygium wilfordii* Hook F, has been proven to inhibit heat shock protein 90 (HSP90) activity and has attracted much attention because of its promising effects in cancer treatment and in ameliorating degenerative neuron diseases. However, the HSP90 structure involved in celastrol interaction is not known. Here, we report a novel celastrol‐binding pocket in the HSP90 dimer, predicted by molecular docking. Mutation of the two key binding pocket amino acids (Lys546 and Tyr493) disrupted the binding of celastrol to HSP90 dimers, as detected by isothermal titration calorimetry (ITC). Interestingly, such mutations also reduced binding between HSP90 and the cochaperone Cdc37, thus providing a new explanation for reported findings that celastrol shows more obvious effects in disrupting binding between HSP90 and Cdc37 than between HSP90 and other cochaperones. In short, our work discloses a novel binding pocket in HSP90 dimer for celastrol and provides an explanation as to why celastrol has a strong effect on HSP90 and Cdc37 binding.

**Abbreviations**
Cdk4: cyclin‐dependent kinase4
HSF‐1: heat shock factor‐1
HSP70: heat shock protein 70
Hsp90: heat shock protein 90
ITC: isothermal titration calorimetry

The compound celastrol, an extraction from traditional Chinese medicine *Tripterygium wilfordii*, exhibits inhibitory effects toward several cancer cells of different origins, such as hepatoma carcinoma cells 1, glioblastoma cells 2, lung cancer cells 3, and melanoma cells 4; this action is attributed to HSP90 inhibition. It has been observed that in celastrol's presence, the binding affinities of HSP90 to its client proteins, as well as to cochaperones, decrease with HSP90–Cdc37 binding showing the most significant decrease 5. It has been proven that celastrol can directly bind to HSP90 and thus disrupt the association of HSP90 and Cdc37 6; yet the chemical basis for celastrol's interaction with HSP90 remains unclear.

It has been about 10 years since celastrol's ability to inhibit HSP90 was discovered, and the understanding of how this inhibition takes place has since developed: First, Westerheide *et al*. 7 reported that celastrol could induce heat shock response by activating HSF‐1, providing indirect evidence that celastrol could inhibit HSP90, as HSF‐1 is always activated when HSP90 is inhibited. Then, by comparing gene expression signatures among different agents, Hieronymus *et al*. 8 found that celastrol inhibits HSP90 by binding to a pocket other than the known ATP‐binding site in HSP90's N terminus, meaning celastrol is a novel type of HSP90 inhibitor. Zhang *et al*. 5 then reported that celastrol disrupted HSP90–Cdc37 interaction in the super chaperone complex to exhibit antitumor activity *in vitro* and *in vivo*, with the molecular docking studies demonstrating that celastrol disrupts a critical interaction between the Glu33 side chain of HSP90 N terminus and Arg167 side chain of Cdc37. However, Sreeramulu *et al*. 9, via NMR technology, found that celastrol does not directly bind to the N‐terminal ATP‐binding pocket of HSP90, but rather targets Cdc37 through a nucleophile reaction. Consistent with this finding, in another study, Zhang *et al*. 6, who had previously suggested that celastrol targets the HSP90/Cdc37 complex in the N terminus of HSP90, reported that celastrol directly binds to the C‐terminal region to disrupt HSP90–Cdc37 binding. That celastrol can directly affect HSP90 is also supported by our findings that celastrol could reduce HSP90's intrinsic ATP activity 10. While much more is now understood about celastrol's actions, the chemical basis behind celastrol's interaction with HSP90 has not yet been fully explained.

In this work, we used molecular docking analysis and found a potential celastrol‐binding pocket on HSP90 dimer in the nucleotide‐free apo form. We then mutated two amino acids key to HSP90–celastrol binding at this site. Through isothermal titration calorimetry (ITC) experiments, we found that celastrol can bind to wild‐type, but not mutant HSP90. Interestingly, our results also indicate that these two amino acids are important to binding between Cdc37 and HSP90, by observing the direct effect of celastrol on wild‐type or mutant HSP90 complex in cell‐free system.

## Results and Discussion

Molecular docking studies are a powerful tool to discover the potential binding sites of proteins to their ligands, but such studies rely heavily on the initial model's accuracy. Recent progress in HSP90 structure analysis has confirmed that HSP90 forms dimers, and that the dimeric conformation changes dramatically along the ATP‐driven HSP90 cycle. Four types of HSP90 dimeric structures have been suggested: Nucleotide‐free Apo, Compact extended ATP (HSP90: C: ATP), Closed (HSP90: C: ATP/ADP+Pi), and Extended ADP‐bound (HSP90: ADP) 11. We thought previous studies' inability to identify the binding sites on HSP90 might be due to a lack of accurate information about HSP90's structure, so for this study we reworked molecular docking studies for celastrol–HSP90 binding using all four of the above conformations.

**Figure 1:**
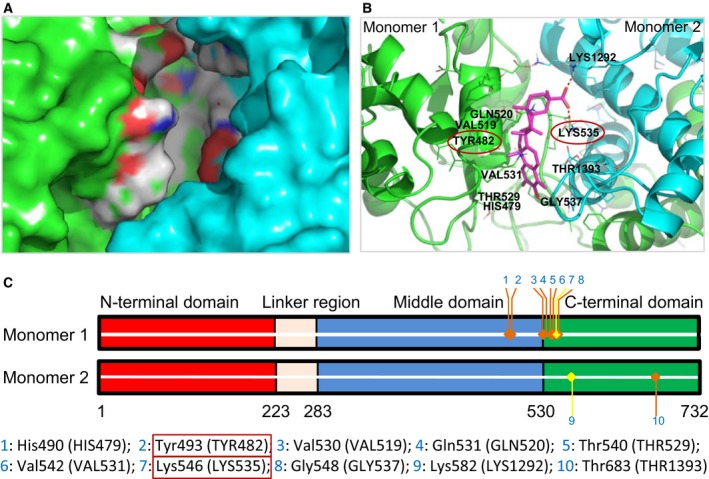
Binding of celastrol to HSP90α dimer by molecular docking. (A) Celastrol's binding pocket in the HSP90α dimer. (B) The amino acids comprising the celastrol‐binding pocket on HSP90α dimer. The mutated amino acids are circled. (C) HSP90α dimer scheme. 1–10 refer to the amino acid sites in celastrol's binding pocket.

Our molecular docking studies demonstrated that in the Nucleotide‐free Apo conformation, a celastrol‐binding pocket consisting of a C‐terminal segment from one monomer and the middle region from another monomer was identified. The cavity was about 18.0 Å in length and 8.0 Å in depth. When situated in this cavity, the two oxygen atoms of celastrol's carboxyl group could form two salt bridges, one with Lys546 (LYS535) (from one monomer) and one with Lys582 (LYS1292) (from another monomer). These two bridges enhanced the binding between celastrol and HSP90 dimers (Fig. 1).

**Figure 2:**
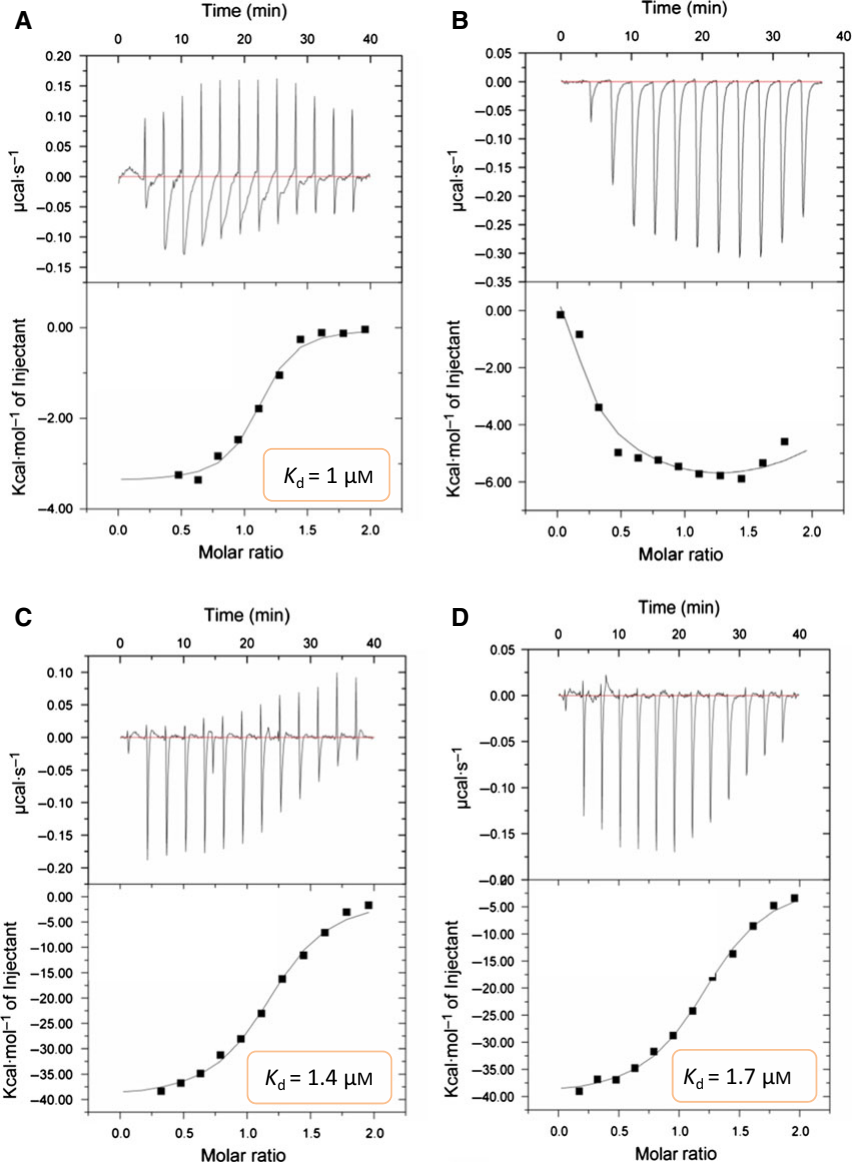
Binding of celastrol or 17‐AAG to wild or mutant human HSP90α, identified by ITC. Celastrol and wild HSP90α (A) or mutant HSP90α (B), tested by ITC (1 : 10 mixture of celastrol/HSP90α). 17‐AAG and wild HSP90α (C) or mutant HSP90α (D), tested by ITC (1 : 10 mixture of 17‐AAG/HSP90α).

**Figure 3:**
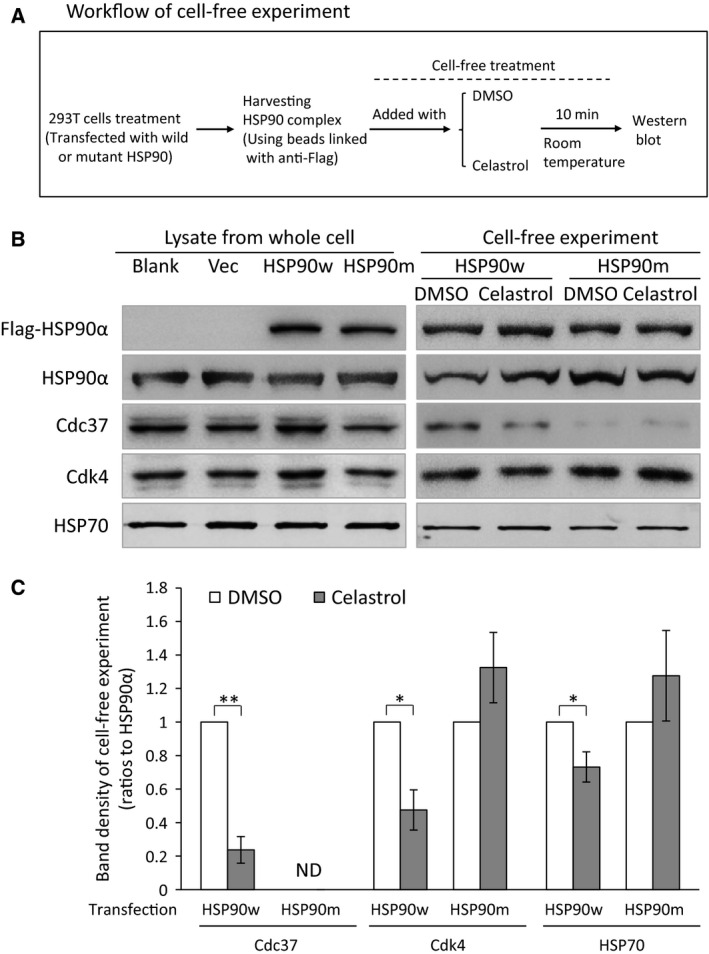
Effects of celastrol on wild‐type or mutant HSP90 complex in cell‐free system. (A) The flowchart for cell‐free experiment. The cells transfected with wild or mutant HSP90 were lysed and the whole proteins were extracted. Wild or mutant HSP90 complexes were captured by incubation of protein extracts with Protein A/G plus agarose and anti‐Flag antibody. The beads with HSP90 complex were incubated with celastrol or DMSO for 10 min before being heated to collect proteins. The proteins were subjected to western blot. (B) Western blot of the related proteins. The left panels show proteins from whole cells, while the right panels show proteins harvested from beads after cell‐free treatment. (C) The histogram and statistical results of band densities from western blot of proteins in beads after cell‐free treatments (as shown in the right panel of B). Band densities of Cdc37, Cdk4, and HSP70 were adjusted using that of HSP90α bands, and the calibrated value of each protein from the DMSO group was set as 1.0. Blank: cells without transfection. Vec: cells transfected with vector. HSP90w: cells transfected with HSP90–wild plasmid. HSP90m: cells transfected with HSP90–mutant plasmid. ND: densities were not detectable by scanning. The data are presented as mean ± standard deviation, *n* = 3. *P < 0.05; **P < 0.01.

To verify the model, we prepared mutant K546D (disrupting one of the predicated salt bridges) and Y493G (damaging one wall of the celastrol‐binding cavity). Using ITC, we found that the mutant proteins lost their ability to bind to celastrol (Fig. 2). On the contrary, both wild and mutant HSP90 could bind to 17‐AAG, which is known to bind to the ATP‐binding site in HSP90's N terminus 12, 13. Through cell‐free experiments with wild or mutant HSP90 complex coimmunoprecipitated with beads, we additionally found that the mutant proteins had reduced ability to bind to Cdc37 while retaining the ability to bind with Cdk4 and HSP70 (Fig. 3). Moreover, celastrol could reduce binding between HSP90 and Cdc37, Cdk4, and HSP70 in wild HSP90 complex, while such effects disappeared in the mutant HSP90 complex (Fig. 3B,C), providing additional evidence that the mutant sites are important to celastrol's effects on HSP90 function.

Our findings are consistent with previous reports that celastrol interacts with HSP90's C terminus 6, 14. Mutation in the celastrol‐binding region reduced HSP90's ability to bind to Cdc37, indicating that this region is important for Cdc37 binding. This is also consistent with the belief that celastrol's more prominent effects result from disruption of HSP90/Cdc37 binding 5.

## Conclusions

In conclusion, based on molecular docking, ITC determination, and the observed effects on HSP90 complex, our work provides novel evidence that celastrol can directly bind to HSP90 dimers, and that some residues in the C terminus of one monomer and in the middle region of another monomer of HSP90 dimers formed a celastrol‐binding pocket. Two oxygen atoms from celastrol's carboxyl group could form two salt bridges, one with Lys546 and one with Tyr493 on HSP90. This celastrol‐binding pocket is the key to HSP90–Cdc37 binding.

## Experimental procedures

### Molecular docking

HSP90 dimers may exist in four states, i.e., nucleotide‐free apo, ATP‐bound compact extended, ATP‐bound closed, and ADP‐bound extended states. The coordinates for each of these models were generous gifts from R.L. Matts (Oklahoma State University), whose group recently constructed these models 11. Initial inspection of the three‐dimensional structures in full‐length HSP90 dimer in the four above states suggests that the nucleotide‐free apo form is most likely to bind to the celastrol molecule; the other forms do not provide cavities large enough for small molecule binding in either the M‐ or C‐terminal domain. Thus, the nucleotide‐free apo form of HSP90 was used for our molecular docking studies with celastrol.

Molecular docking was carried out using Schrodinger Suite 2009. The ligand (celastrol) was prepared using the ligprep module, and the dimeric molecules of full‐length HSP90 were preprocessed using the protein preparation wizard before docking. The prepared ligand was docked into the nucleotide‐free apo form of HSP90 using the Glide module at standard precision, and the conformation with the best docking score was selected as our starting model for subsequent induced fit docking (IFD). IFD was carried out using the suite's IFD workflow, in which side chains within 5 Å of celastrol were softened and refined during induced fit docking. No positional or hydrophobic constraint was used.

### Plasmid construction and site‐directed mutagenesis

Prokaryotic expression plasmid pET15b‐hHSP90α for human full‐length His‐HSP90α protein was kindly gifted by T. Ratajczak (University of Western Australia, Australia). By cloning, HSP90α (NCBI accession number: NP_005339) was inserted into prokaryotic or eukaryotic expression vector pSUMO‐Mut or pCMV‐tag2a for site‐directed mutagenesis and protein expression or for transient transfection (Fig. S1).

Mutants containing two sites (Y493G and K546D) of HSP90α were created by QuickChange kit (Stratagene, Santa Clara, CA, USA), and sequenced to confirm the correct incorporation of the mutations.

### Protein expression and purification

Wild‐type human HSP90α and mutant proteins were expressed in *E. coli* strain BL21 (DE3) and purified by nickel affinity chromatography. After the purification, the proteins were dissolved in a buffer containing 10 mm Hepes pH 7.4, 150 mm NaCl, and 0.5 mm EDTA, and stored at −80 °C. The purified proteins were confirmed by gel electrophoresis (Fig. S2).

### Isothermal titration calorimetry

The purified wild or mutant HSP90α proteins were dialyzed against in the buffer containing 10 mm Hepes pH 7.4, 150 mm NaCl, and 0.5 mm EDTA using Slide‐A‐Lyzer^™^Dialysis Cassettes (Thermo Fisher Scientific Inc., Rockford, lL, USA) three times 15. Then, the dialyzed proteins were loaded with celastrol or DMSO by MicroCal iTC200 (GE Healthcare, Marlborough, MA, USA). The cells were thermostated at 25 °C. The reaction between wild or mutant HSP90α proteins and celastrol was conducted with 60 μm protein in the cell and 600 μm ligand in the syringe, while the reaction between proteins and 17‐AAG was performed with 30 μm protein in the cell and 600 μm ligand in the syringe. The experiment was conducted with 13 injections, with a volume of 3 μL and 180 s spacing.

### Transient transfection, coimmunoprecipitation, cell‐free treatment, and western blot

Transient transfection was performed into 293T cells according to the manufacturer's instructions (lipofectamine 2000; Invitrogen, Carlsbad, CA, USA).

For coimmunoprecipitation, transfected cells were incubated in lysis buffer (20 mm Tris/HCl, 25 mm NaCl, 0.1% NP40, 2 mm DTT, 20 mm Na_2_MoO_4_, and protease inhibitor cocktail, pH 7.4). Two milligrams of proteins were incubated with 4 μg of anti‐Flag antibody, and then 60 μL Protein A/G plus agarose was added. Beads were washed three times with PBS.

The wild or mutant HSP90 complexes captured by agarose beads were used for cell‐free treatment. The beads were divided into two groups, and incubated with DMSO or celastrol (final concentration of 12.5 μm) in PBS, respectively. After reaction at room temperature for 10 min, the beads were heated and the proteins collected for western blot, which was carried out according to routine protocol.

## Author contributions

BP carried out ITC and cell experiments, participated in the design of the study, and drafted the manuscript. YG carried out molecular docking and participated in drafting the manuscript. YW carried out western blot. FC participated in cell culture. XZ carried out ITC. DZ and JH conceived of the study, participated in its design and coordination, and helped to draft the manuscript. All authors have read and approved the final manuscript.

## Supporting information


**Fig. S1.** Construction and gel verification of mutant or wild HSP90 expression vector.
**Fig. S2.** Protein expression and purification.
