# Supplementary material for: Mutations Y493G and K546D in human HSP90 disrupt binding of celastrol and reduce interaction with Cdc37
Source: FEBS Open Bio. 2016 May 25;6(7):729–34. doi: 10.1002/2211-5463.12081 (PMC4932452; doi:10.1002/2211-5463.12081)
Supplement: Supplementary file 1 — Fig. S1. Construction and gel verification of mutant or wild HSP90 expression vector. Fig. S2. Protein expression and purification. [file FEB4-6-729-s001.docx]

Supplementary file

**Construction and gel verification of mutant or wild HSP90 expression vector**

As shown in Figure S1, HSP90 wild or mutant gene sequences were inserted into the pCMV-Tag2 vector, adjoining and down-stream of the Flag. Thus, the expressed wild or mutant HSP90 proteins were flagged with a Flag tag.

**
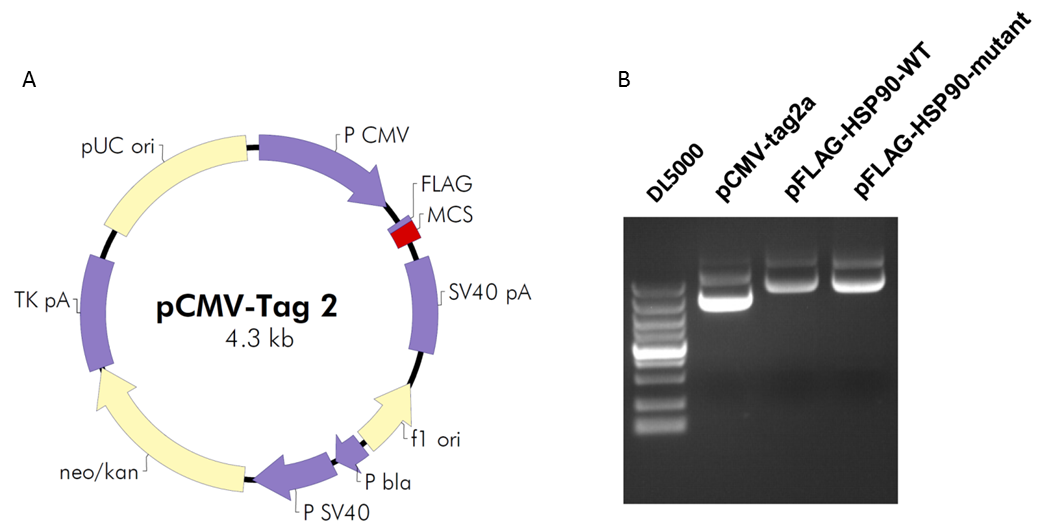
**

**Figure S1**. The vector map of pCMV-Tag2 (*A*) and the gel results of Flag-HSP90-WT and mutant plasmids (*B*).

**Protein expression and purification**

Wild-type human HSP90α and mutant proteins were expressed in E. coli strain BL21 (DE3) and purified by nickel affinity chromatography. After the purification, the proteins were dissolved in a buffer containing 10 mM Hepes pH7.4, 150 mM NaCl, and 0.5 mM EDTA, and stored at -80°C. The purified proteins were confirmed by gel electrophoresis.

**
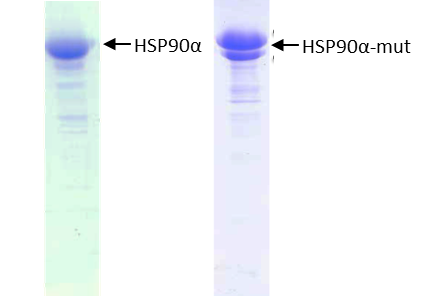
**

**Figure S2**. Protein gels showing the purity of purified proteins
